# Downregulation of NDUFB6 due to 9p24.1-p13.3 loss is implicated in metastatic clear cell renal cell carcinoma

**DOI:** 10.1002/cam4.351

**Published:** 2014-10-15

**Authors:** Takahiro Narimatsu, Keiko Matsuura, Chisato Nakada, Yoshiyuki Tsukamoto, Naoki Hijiya, Tomoki Kai, Toru Inoue, Tomohisa Uchida, Takeo Nomura, Fuminori Sato, Masao Seto, Ichiro Takeuchi, Hiromitsu Mimata, Masatsugu Moriyama

**Affiliations:** 1 Department of Molecular Pathology, Faculty of Medicine, Oita UniversityOita, Japan; 2 Department of Urology, Faculty of Medicine, Oita UniversityOita, Japan; 3 Divison of Molecular Medicine, Aichi Cancer Center Research InstituteNagoya, Japan; 4 Department of Computer Science/Scientific and Engineering Simulation, Nagoya Institute of TechnologyNagoya, Japan

**Keywords:** 9p, array CGH, kidney cancer, metastasis, NDUFB6

## Abstract

This study was conducted to clarify the genomic profiles of metastatic clear cell renal cell carcinomas (ccRCCs) and identify the genes responsible for development of metastasis. We analyzed the genomic profiles of 20 cases of primary ccRCC and their corresponding metastases using array-based comparative genomic hybridization, and identified 32 chromosomal regions in which gene copy number alterations were detected more frequently in metastases than in the primary tumors. Among these 32 regions, 9p24.1-p13.3 loss was the most statistically significant alteration. Furthermore, we found that patients with 9p24.1-p13.3 loss in primary tumors exhibited significantly lower rates of recurrence-free and cancer-specific survival, suggesting that 9p loss in the primary tumor is a potential biomarker predicting early recurrence of metastasis. Interestingly, the genomic profiles of primary tumors with 9p loss resembled those of their corresponding metastases, though 9p loss was accumulated in the metastases derived from the primary tumors without 9p loss. Comparison of the mRNA expression levels revealed that 2 of 58 genes located at 9p24.1-p13.3 were downregulated due to gene copy number loss in ccRCCs. An overexpression study of these two genes in ccRCC cell lines revealed that downregulation of *NDUFB6* due to loss at 9p24.1-p13.3 may confer a growth advantage on metastatic ccRCC cells. These results were confirmed by analyzing the data of 405 cases of ccRCC obtained from The Cancer Genome Atlas (TCGA). On the basis of our present data, we propose that *NDUFB6* is a possible tumor suppressor of metastatic ccRCCs.

## Introduction

Inactivation of the von Hippel-Lindau (VHL) gene whose protein product acts as an E3 ubiquitin ligase targeting the transcription factor, hypoxia-inducible factor-*α* (HIF-*α*), is known to occur in almost all cases of clear cell renal cell carcinoma (ccRCC) 1–3. Therefore, it is believed that abnormal activation of the VHL-HIF-*α* signaling pathway plays an essentially important role in ccRCC carcinogenesis, and that the signaling molecules comprising this pathway would be potential candidates for molecular-targeted chemotherapy for ccRCC patients.

However, molecular mechanisms involving ccRCC metastasis are still poorly understood. It has already been reported that gene copy number alterations (CNAs) including 9p loss are detected more frequently in metastatic lesions than in primary tumors 4–6. It has also been reported that 9p loss is detected more frequently in primary lesions of patients who develop metastases at an early stage 7–11. In order to elucidate the deregulated signaling pathway responsible for the proliferation and survival of metastatic ccRCC cells, we carried out genomic profiling of metastatic ccRCC cells using array-based comparative genomic hybridization (array CGH). By comparing the primary tumors with their corresponding metastatic tumors in each patient, we identified 9p24.1-p13.3 loss as the CNAs most frequently detected in metastases. Furthermore, within 9p24.1-p13.3, we tried to identify candidate tumor suppressor genes that were significantly downregulated due to gene copy number loss.

## Materials and Methods

### Patients

All the study samples were obtained by surgical resection at Oita University Hospital and Oita Red Cross Hospital and diagnosed histopathologically as described previously 12. Formalin-fixed, paraffin-embedded (FFPE) samples of 20 primary ccRCCs and their corresponding metastatic lesions were used for array CGH. We defined this group as patient group I (Table S1). Another set comprising 30 frozen specimens of primary ccRCCs were also used for array CGH and quantitative RT-PCR. For 19 of these 30 cases, frozen samples of normal renal cortex tissue as well as ccRCC tissue were also obtained. We defined this group as patient group II (Table S1). The clinical courses of all patients in group I are shown in Figure S1. All samples of metastases were obtained from distant locations, and not from regional lymph nodes (Fig. S1). The use of the tissue samples for all experiments was approved by the Oita University Ethics Committee (Approval no. P-05-05) in accordance with the Ethical Guidelines for Clinical Research of the Japanese Ministry of Health, Labour, and Welfare 2008 (http://www.mhlw.go.jp/english/).

### DNA extraction

Genomic DNA for array CGH was extracted from FFPE tissue sections of the 20 primary ccRCCs and their corresponding metastatic lesions (patient group I), as described previously 13. As a control, genomic DNA was extracted from FFPE samples of normal renal cortex obtained from each patient. Genomic DNA for array CGH was also extracted from frozen tissue sections of 30 cases of primary ccRCC (patient group II), as described previously 12. The same amount of genomic DNA extracted from peripheral blood cells of eight healthy male volunteers was mixed and used as control DNA for the frozen tumor samples, as described previously 14. All tumor samples included at least 70% tumor cells by histologic examination.

### Array CGH and data analysis

Array CGH was performed using the Agilent Whole Human Genome 4x44 K Oligo Micro Array Kit (Agilent Technologies Inc., Santa Clara, CA) in accordance with the manufacturer's instructions. The data obtained in the array CGH analysis are available on the GEO database (http://www.ncbi.nlm.nih.gov/geo/; accession number GSE 43477). More details of the method used for the array CGH analysis are described in Data S1.

### RNA extraction and quantitative RT-PCR

Total RNA was extracted from 30 primary ccRCCs (patient group II) and 19 samples of normal renal cortex using an RNeasy mini kit (Qiagen, Valencia, CA). Quantitative RT-PCR was performed with a Universal probe library (Roche Diagnostics, Mannheim, Germany) and a LightCycler 480 probe master (Roche Diagnostics) by the Taqman method, as described previously 14. Messenger RNA expression levels relative to KPNA6 were obtained from a standard curve, and normalized to the median value for 19 samples of normal renal cortex. *P* values were calculated by the Kruskal–Wallis test and Steel–Dwass test as a post hoc test.

### Cell culture

The RCC cell lines 786-O (#CRL-1932) and 769-P (#CRL-1933) were purchased from the American Type Culture Collection (ATCC) (Rockville, MD) and maintained in RPMI1640 supplemented with 10% fetal bovine serum.

### Lentiviral vector production and in vitro transduction

The full-length *NDUFB6* and *LRRC19* cDNA was obtained from NBRC (NITE Biological Resource Center, Chiba, Japan) and cloned into pLenti7.3/V5-DEST (Invitrogen, Carlsbad, CA) using the Gateway system™ in accordance with the manufacturer's instructions. Lentiviruses encoding *NDUFB6* (Lv-NDUFB6), *LRRC19* (Lv-LRRC19), and no cDNAs (Lv-Control) were produced as described previously 14, and were used for the transduction experiment. Each lentivirus was added to 786-O and 769-P cells and incubated for 24 h with a transducing unit of 1 using Polybrene at a final concentration of 6.0 *μ*g/mL. Transduced cells were then subjected to proliferation, apoptosis, and invasion assay.

### siRNA transfection

Cells were transfected with Stealth™ RNAi oligonucleotide or the Stealth™ RNAi Negative Control Duplex with the corresponding GC content (Invitrogen) at a final concentration of 2 nmol/L using Lipofectamine RNAiMAX (Invitrogen) in accordance with the manufacturer's instructions. The target sequences of siRNAs are as follows: NDUFB6 siRNA1, 5′-TCATGTACTTGTACCTGTCTGGATT-3′; NDUFB6 siRNA2, 5′-GAGCCTAAGTTTGTTCCTATATTAC-3′.

### Western blot analysis

Western blot analysis was performed in a similar way to our previous study 15. In each experiment, 10–20 *μ*g of cell lysate was subjected to analysis. The primary antibodies employed were anti-NDUFB6 antibody (for overexpression: Sigma-Aldrich, St. Louis, MO; for knockdown: Atlas Antibodies AB, Stockholm, Sweden), anti-human GAPDH antibody (Sigma-Aldrich), and anti-V5 antibody (Bethyl Laboratories Inc., Montgomery, TX). Detection was performed with ECL Prime Western Blotting Detection Reagents (GE Healthcare, Piscataway, NJ) in accordance with the manufacturer's instructions.

### Proliferation assay

After infection of Lv-NDUFB6, Lv-LRRC19, or Lv-Control to 786-O and 769-P cells in a 96-well plate, MTS assay was carried out using the CellTiter 96^**®**^ AQ_ueous_ One Solution Cell Proliferation Assay Kit (Promega, Madison, WI), and the absorbance at 492 nm (reference wave length 690 nm) was measured using a fluorescence reader (Multiskan GO) (Thermo Scientific, Waltham, MA) in accordance with the manufacturer's instructions. Each condition was reproduced in quadruplicate, and the experiment was performed twice. MTS assay was also carried out after transfection of siRNA to 786-O and 769-P in a 96-well plate.

### TCGA dataset analysis

The results published here are in whole or part based on data generated by The Cancer Genome Atlas (TCGA) Research Network (http://cancergenome.nih.gov/). Data of genomic alterations including mRNA expression and DNA copy number for 405 of 499 ccRCCs were obtained from cBioPortal for Cancer Genomics (http://www.cbioportal.org/). Clinical information including recurrence-free survival was extracted from TCGA dataset.

### Statistical analysis

McNemar test, log-rank test, Fisher's exact test, Kruskal–Wallis test, Steel–Dwass test, Mann–Whitney *U* test, and paired *t*-test were used. Differences at *P *<* *0.05 were considered statistically significant.

## Results

### CNAs are more frequently detectable in metastases than in primary tumors

**Figure 1:**
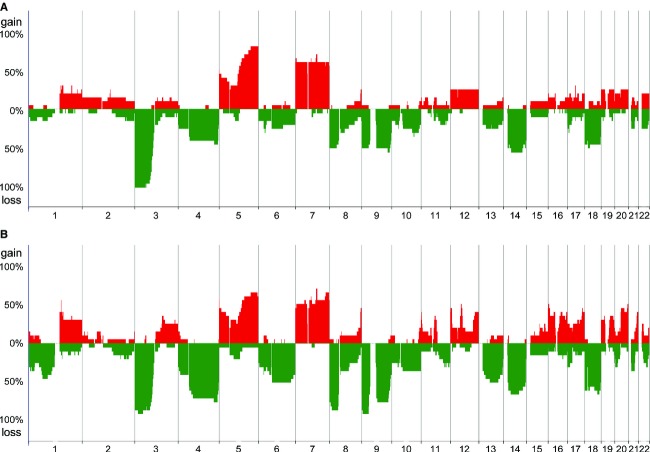
Genomic profiles of primary ccRCCs and corresponding metastases. Genome-wide averaged frequencies of genomic imbalances in 20 cases (patient group I) of primary ccRCC (A) and their corresponding metastases (B). Oligonucleotide probes are shown in order within each chromosome from chromosomes 1 to 22. The frequencies (%) of gains (positive axis) and losses (negative axis) are shown for each probe in primary ccRCCs and their corresponding metastases.

Using array CGH, we first analyzed FFPE samples of 20 primary ccRCCs and their corresponding metastases, and compared the CNAs of the primary tumors with those of the metastases (patient group I). We found that the genomic profiles of the metastases resembled those of the primary tumors as a whole (Fig.1). Using the McNemar test, we compared the CNAs in the two groups, and identified 13 CNAs that were detected more frequently in metastases (Table1). Among the 13 CNAs, we identified 32 chromosomal regions in which CNAs were more frequent in metastases (McNemar test, *P *<* *0.05, Table1).

**Table 1:** CNAs frequently detected in metastases.

Genomic CNAs								
Gain/Loss	Chromosomal region (bp)^7^		Size (bp)	Primary ccRCCs^1^ (*n* = 20) (%)	Metastases^2^ (*n* = 20) (%)	McNemar test (*P* value)	Recurrence-free survival^3^ (*P* value)	Cancer-specific survival^4^ (*P* value)
Gains								
1q21.1	143,706,582–143,787,563	(1q21.1)	80,981	2 (10)	8 (40)	0.031	NS	NS
11p15.5	274,838–305,091	(11p15.5)	30,253	1 (5)	7 (35)	0.031	NS	NS
16p13.3	3,346,012–4,759,330	(16p13.3)	1,413,318	3 (15)	10 (50)	0.039	NS	NS
	4,904,632–4,904,686	(16p13.3)	54	3 (15)	10 (50)	0.039	NS	NS
Losses								
1p32.1-p31.3	60,310,897–64,442,244	(1p32.1-p31.3)	4,131,347	2 (10)	8 (40)	0.031	NS	NS
1p31.3-p21.1	64,788,851–86,944,864	(1p31.3-p22.3)	22,156,013	3 (15)	9 (45)	0.031	NS	NS
	86,968,324–86,968,383	(1p22.3)	59	2 (10)	9 (45)	0.016	NS	NS
	87,017,943–104,653,907	(1p22.3-p21.1)	17,635,964	2 (10)	8 (40)	0.031	NS	NS
4q13.3	70,439,721–75,081,744	(4q13.3)	4,642,023	8 (40)	14 (70)	0.031	NS	NS
4q13.3-q35.1	75,122,238–167,949,936	(4q13.3-q32.3)	92,827,698	8 (40)	14 (70)	0.031	NS	NS
	168,158,098–169,858,273	(4q32.3)	1,700,175	8 (40)	15 (75)	0.016	NS	NS
	169,986,365–182,690,789	(4q32.3-q35.1)	12,704,424	9 (45)	15 (75)	0.031	NS	NS
4q35.2	187,416,579–188,522,372	(4q35.2)	1,105,793	5 (25)	12 (60)	0.016	0.0455^5^	NS
	189,694,932–190,351,920	(4q35.2)	656,988	4 (20)	10 (50)	0.031	0.0461^5^	NS
	190,887,201–191,121,254	(4q35.2)	234,053	3 (15)	9 (45)	0.031	NS	NS
6q22.1-q22.31	114,483,498–119,876,171	(6q22.1-q22.31)	5,392,673	4 (20)	10 (50)	0.031	NS	NS
6q22.31-q25.1	121,268,217–150,251,493	(6q22.31-q25.1)	28,983,276	4 (20)	10 (50)	0.031	NS	NS
8p23.1	10,323,367–10,323,426	(8p23.1)	59	9 (45)	16 (80)	0.039	NS	NS
8p23.1-p11.21	11,721,042–31,607,604	(8p23.1-p12)	19,886,562	10 (50)	17 (85)	0.016	NS	NS
	31,616,802–33,525,017	(8p12)	1,908,215	9 (45)	17 (85)	0.008	NS	NS
	33,568,440–33,575,306	(8p12)	6866	9 (45)	16 (80)	0.039	NS	NS
	34,072,415–36,481,098	(8p12)	2,408,683	9 (45)	17 (85)	0.008	NS	NS
	36,822,238–36,912,314	(8p12)	90,076	9 (45)	16 (80)	0.039	NS	NS
	36,973,293–38,988,375	(8p12-p11.23)	2,015,082	9 (45)	17 (85)	0.008	NS	NS
	39,018,630–40,131,529	(8p11.23-p11.21)	1,112,899	9 (45)	16 (80)	0.016	NS	NS
	40,208,325–40,802,376	(8p11.21)	594,051	8 (40)	15 (75)	0.039	NS	NS
9p24.3-p13.3	2,078,528–2,078,587	(9p24.3)	59	9 (45)	16 (80)	0.039	0.0196^6^	0.0036^6^
	2,109,502–2,822,760	(9p24.3-p24.2)	713,258	9 (45)	17 (85)	0.021	0.0196^6^	0.0036^6^
	2,906,200–5,500,754	(9p24.2-p24.1)	2,594,554	10 (50)	17 (85)	0.039	0.0089^6^	0.0006^6^
	5,536,660–32,972,853	(9p24.1-p13.3)	27,436,193	10 (50)	18 (90)	0.008	0.0089^6^	0.0006^6^
	32,978,063–33,019,061	(9p13.3)	40,998	10 (50)	17 (85)	0.039	0.0089^6^	0.0006^6^
	33,027,699–34,076,033	(9p13.3)	1,048,334	9 (45)	16 (80)	0.016	0.0196^6^	0.0036^6^

Underlining indicates the CNA for which the frequency of metastasis was the most significant relative to the primary tumor and associated with poor prognosis.

1 Primary ccRCCs in which each CNA was detected.

2 Metastases in which each CNA was detected.

3 Comparison of recurrence-free survival rates between patients with and without CNAs in their primary ccRCCs by log-rank test.

4 Comparison of cancer-specific survival rates between patients with and without CNAs in their primary ccRCCs by log-rank test.

5 Patients without CNAs showed lower recurrence-free survival rates than the patients with CNAs.

6 CNAs that were associated with significantly lower recurrence-free survival rates and cancer-specific survival rates if detected in the primary tumors.

7 Genomic locations are based on the human reference genome UCSC hg18 assembly.

### Poor prognosis of patients with 9p24.1-p13.3 loss

**Figure 2:**
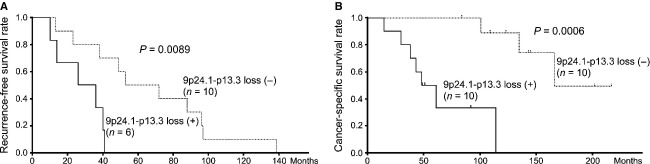
Poor prognosis of patients with 9p24.1-p13.3 loss in primary ccRCCs. (A) Recurrence-free survival rates (shown by Kaplan–Meier curve) for six patients with 9p24.1-p13.3 loss in primary ccRCCs, excluding four patients with stage IV disease, were lower than those for 10 patients without 9p24.1-p13.3 loss (*P *= 0.0089 determined by log-rank test). (B) Cancer-specific survival rates (shown by Kaplan–Meier curve) for 10 patients with 9p24.1-p13.3 loss in primary ccRCCs were lower than those for 10 patients without 9p24.1-p13.3 loss (*P *=* *0.0006 determined by log-rank test).

Next, among the 32 chromosomal regions in which CNAs were more frequent in metastases, we tried to identify the CNAs associated with poor prognosis. Therefore, we divided the 20 patients (patient group I) into two groups based on whether or not their primary tumors had each of the CNAs that were frequently detectable in metastases. For each of the 32 chromosomal regions, the recurrence-free and cancer-specific survival rates were compared between the two groups. Of the 32 chromosomal regions analyzed, patients with loss at 9p24.3-p13.3 including six regions (9p24.3, 9p24.3-p24.2, 9p24.2-p24.1, 9p24.1-p13.3, and 9p13.3) in the primaries showed a significantly lower recurrence-free and cancer-specific survival rates than patients without such loss (Table1). Furthermore, among these six regions, loss at 9p24.1-p13.3 was that most frequently detected in metastases (Table1 and Fig. S2), and was associated with significantly lower rates of recurrence-free and cancer-specific survival when detected in the primary tumor (Table1, Fig.2A and B). We then investigated the recurrence-free and cancer-specific survival rates of the patients including both patient group I and II, and found that loss at 9p24.1-p13.3 in the primary tumor was also associated with poor prognosis (Fig. S3). Based on these results, it is suggested that loss at 9p24.1-p13.3 is the most common CNA associated with poor prognosis among the 32 chromosomal regions. In patient group I, 10 of the 20 patients had been treated by therapeutic agents before surgical resection of metastases (Table S1). No significant differences in cancer-specific survival rates were found between the cases who were administrated therapeutic agents and the cases who were not (Fig. S4). Furthermore, the genomic profiles of the metastases of these two groups were similar to each other (Fig. S5). To avoid the possibility that loss at 9p24.1-p13.3 might be a CNA associated with progression, we compared the frequencies of loss at 9p24.1-p13.3 between locally advanced ccRCCs: T2, T3, or T4 tumors without metastases (*n* = 11) and metastases (*n* = 20). Loss at 9p24.1-p13.3 tended to be detected more frequently in metastases than in locally advanced ccRCCs (*P *=* *0.0665 by Fisher's exact test, data not shown). Therefore, it is concluded that loss at 9p24.1-p13.3 is implicated in metastases.

### Tumors with 9p24.1-p13.3 loss have CNAs associated with metastasis

**Figure 3:**
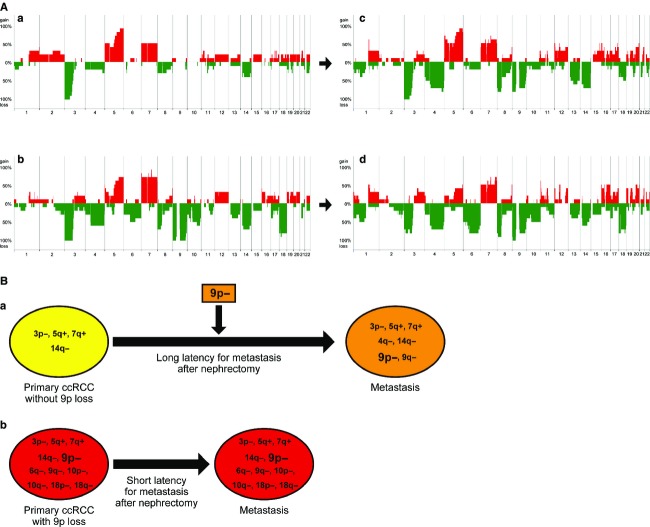
Subclassification of ccRCCs into two groups based on presence or absence of 9p24.1-p13.3 loss. (A) Genome-wide averaged frequencies of genomic imbalances in primary ccRCCs and their corresponding metastases with and without 9p24.1-p13.3 loss. Genome-wide averaged frequencies of genomic imbalances in 10 cases of primary ccRCC without 9p24.1-p13.3 loss (a) and their corresponding metastases (c), as well as 10 cases of primary ccRCC with 9p24.1-p13.3 loss (b) and their corresponding metastases (d), are shown. Oligonucleotide probes are shown in order within each chromosome from chromosomes 1 to 22. The frequencies (%) of gains (positive axis) and losses (negative axis) are shown for each probe. (B) Hypothetical model of metastasis formation in ccRCCs with or without 9p24.1-p13.3 loss. In patients without loss at 9p, it takes a longer time for metastasis to appear after nephrectomy for removal of the primary tumor, and such patients tend to have a relatively good prognosis (a). On the other hand, in patients with loss at 9p accompanied by losses at 6q, 9q, 10p, 10q, 18p, and 18q in the primary tumor, metastasis may occur early, and such patients tend to have a relatively poor prognosis (b).

To investigate the genomic profiles of primary ccRCCs with 9p24.1-p13.3 loss which exhibit poor prognosis, we classified the primary tumors into two groups based on the presence or absence of 9p24.1-p13.3 loss, and compared their genomic profiles. As shown in Figure3A (a), (b), and Table S2, losses at 6q11.1-q22.1, 9p24.3-p24.1, 9p13.3-p13.1, 9q13-q34.13, 9q34.2-q34.3, 10p11.22, 10q21.1-q21.3, 10q21.3-q24.31, 10q24.33-q26.2, 18p11.32-p11.21, and 18q11.1-q23 were detected more frequently in primary tumors with 9p24.1-p13.3 loss than in those without it. This suggests that these CNAs are accompanied by 9p24.1-p13.3 loss.

Remarkably, comparison of genomic profiles between the primary tumors with 9p24.1-p13.3 loss (Fig.3A [b]) and their corresponding metastases (Fig.3A [d]) revealed no significant differences between them (data not shown). This suggests that genomic alterations associated with metastasis may already be present in primary lesions showing 9p24.1-p13.3 loss. This possibility was supported by the finding that patients whose primary lesions showed 9p24.1-p13.3 loss had a significantly poorer outcome (Fig.2). Therefore, in addition to 9p24.1-p13.3 loss in primary tumors, accompanying CNAs detected more frequently in such primaries may also be implicated in metastasis.

On the other hand, we found that the metastases derived from primary tumors without 9p24.1-p13.3 loss acquired several CNAs, including loss at 9p24.3-p13.1 as well as loss at 4q32.3 and 9q13-q33.3 (Fig.3A [a], [c] and Table S3). Losses at 18p and 18q were also acquired, although the difference in their frequency between the primary and the metastasis did not reach statistical significance (Fig.3A [a], [c] and Table S4). This suggests that primary tumors without 9p24.1-p13.3 loss may need to acquire additional CNAs in order to metastasize.

Next, we compared the genomic profiles of metastases whose primary tumors did not have 9p24.1-p13.3 loss (Fig.3A [c]) with those of metastases whose primary tumors showed 9p24.1-p13.3 loss (Fig.3A [d]), and found no significant differences, contrary to our expectation (data not shown).

This suggests that metastases may have genomic losses at 9p24.1-p13.3 together with 6q, 9q, 10p, 10q, 18p, and 18q even if their primary tumors do not have such losses.

### *NDUFB6* and *LRRC19* located at 9p24.1-p13.3 are downregulated due to gene copy number loss

**Figure 4:**
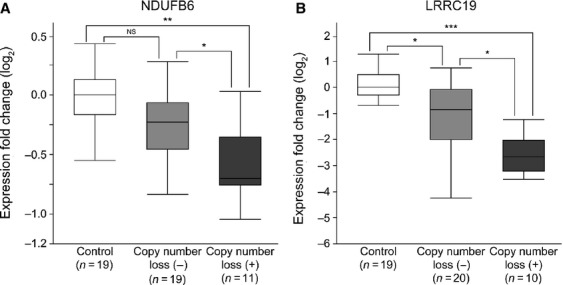
Downregulation of *NDUFB6* and *LRRC19* due to gene copy number loss. Expression levels of *NDUFB6* (A) and *LRRC19* (B) were significantly lower in primary ccRCCs with copy number loss than in those without it, and they were also significantly lower than in normal control tissues. Quantitative RT-PCR analyses of 30 primary ccRCCs and 19 samples of normal control tissue (patient group II) were performed. The *y*-axis displays the expression level (log_2_) normalized by the median expression level for the 19 normal control samples. *P < 0.05, **P <* *0.001, and ***P <* *0.0001 determined by Kruskal–Wallis test and Steel–Dwass test as a post hoc test. NS, not significant.

We hypothesized that the genes located between 5,536,660 and 32,972,853 base pairs (bp) at 9p24.1-p13.3 and downregulated due to gene copy number loss were associated with recurrence or metastasis (Table1). Within this region, 58 annotated genes were assigned on the array CGH platform we employed (Table S5). We then tried to analyze the expression status of these 58 genes. However, we were unable to detect the expression levels of these genes accurately, because only FFPE samples were available for these 20 patients (group I). Therefore, we used another set of patients for whom 30 frozen samples of primary tumors were available (patient group II), and analyzed the expression levels of the 58 genes by quantitative RT-PCR. Next, we analyzed the copy number of each of the 58 genes by array CGH, and divided the patients into two groups based on the presence or absence of copy number loss for each gene (Table S6). Finally, we compared the expression levels of these 58 genes between the two groups. As shown in Figure4, we found that only *NDUFB6* and *LRRC19* were significantly more downregulated in primary tumors with gene copy number loss than in those without it. On the other hand, the expression levels of the other genes including *CDKN2A* and *CDKN2B* showed no significant difference between tumors with and without gene copy number loss (Fig. S6 and Table S5). Based on these data, it is suggested that *NDUFB6* and *LRRC19* are implicated in metastasis.

### NDUFB6 is involved in proliferation of RCC cell lines

**Figure 5:**
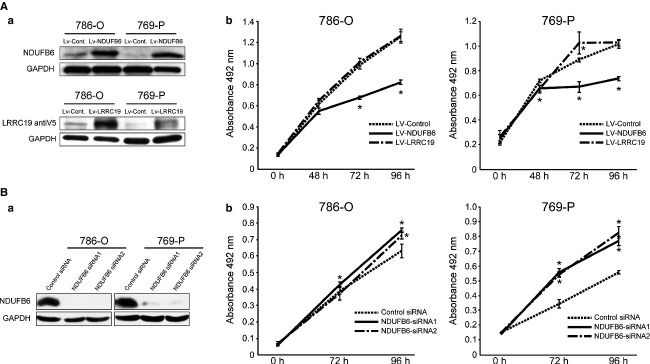
Overexpression of *NDUFB6* suppresses cell proliferation in NDUFB6-downregulated RCC cell lines. (A) Overexpression of *NDUFB6* suppresses the growth of RCC cell lines. (a) Protein expression was confirmed by Western blotting with antibodies against NDUFB6 (Sigma-Aldrich), V5 and GAPDH. (b) MTS assay was carried out at 0, 48, 72, and 96 h after transduction of Lv-Control, Lv-NDUFB6, or Lv-LRRC19 into 786-O cells and 769-P cells. The *y*-axis displays the absorbance at 492 nm (reference wave length 690 nm). The data are expressed as mean ± SEM of quadruplicate determinations, and differences were analyzed by Mann–Whitney *U* test. Two independent experiments were performed and representative data are shown. *P <* *0.05 determined by Mann–Whitney *U* test. (B) Knockdown of NDUFB6 increases cell proliferation of RCC cell lines. (a) The knockdown of protein expression was confirmed by Western blotting with antibodies against NDUFB6 (Atlas Antibodies AB) and GAPDH. (b) MTS assay was carried out at 0, 72, and 96 h after transfection of Stealth™ RNAi oligonucleotide NDUFB6 siRNA1, NDUFB6 siRNA2, or Stealth™ RNAi Negative Control Duplex into 786-O cells and 769-P cells. The *y*-axis displays the absorbance at 492 nm (reference wave length 690 nm). The data are expressed as mean ± SEM of quadruplicate determinations, and differences were analyzed by Mann–Whitney *U* test. Two independent experiments were performed and representative data are shown. *P <* *0.05 determined by Mann–Whitney *U* test.

**Figure 6:**
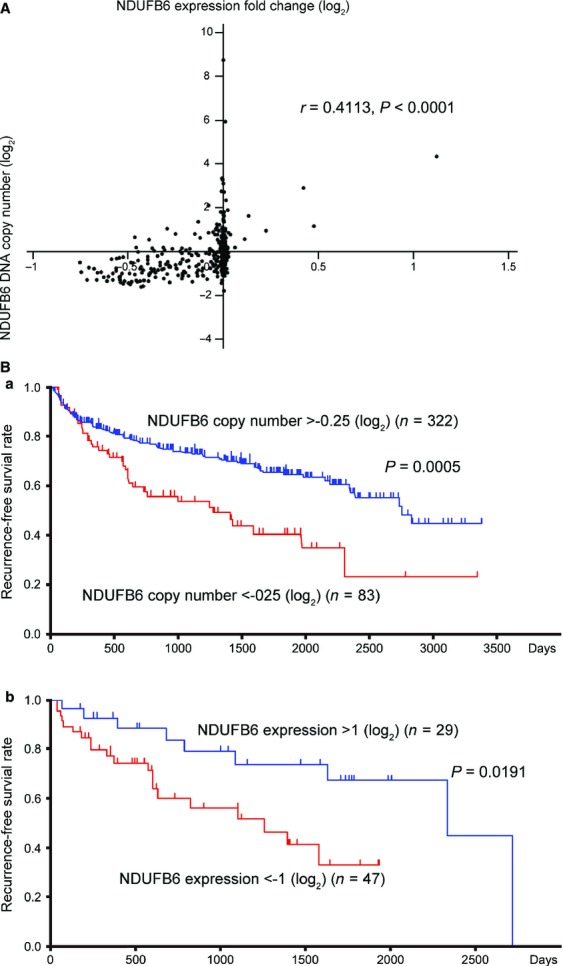
Copy number loss and downregulation of *NDUFB6* are associated with poor prognosis by analyzing TCGA dataset. (A) *NDUFB6* DNA copy number correlates with its mRNA expression. In 405 cases of ccRCC obtained from the TCGA dataset, *NDUFB6* DNA copy number correlated with its mRNA expression level (Pearson correlation coefficient = 0.4113, *P *<* *0.0001). The *x*-axis displays the *NDUFB6* DNA copy number (log_2_). The *y*-axis displays the *NDUFB6* expression fold change (log_2_). (B) Lower *NDUFB6* DNA copy number and mRNA expression level are associated with poor prognosis. (a) Recurrence-free survival rates (shown by Kaplan–Meier curve) for 83 cases obtained from TCGA dataset who showed *NDUFB6* DNA copy number less than −0.25 (log_2_), were lower than those for 322 cases who showed more than −0.25 (log_2_) (*P *=* *0.0005 determined by log-rank test). (b) Recurrence-free survival rates (shown by Kaplan–Meier curve) for 47 cases obtained from TCGA dataset who showed *NDUFB6* expression fold change less than −1.0 (log_2_) were lower than those for 29 cases who showed more than 1.0 (log_2_) (*P *=* *0.0191 determined by log-rank test).

We investigated whether overexpression of NDUFB6 and LRRC19 may affect the proliferation of the RCC cell lines 786-O and 769-P, in which the expression levels of these genes are downregulated (Fig. S7). We found that cell proliferation was suppressed by transduction of Lv-NDUFB6, but was unchanged by transduction of Lv-LRRC19 (Fig.5A). Furthermore, we analyzed the populations of apoptotic cells after transduction of Lv-NDUFB6 or Lv-LRRC19 in both cell lines, but no significant differences were evident (Fig. S8). Then, we investigated whether downregulation of NDUFB6 is implicated in cell proliferation. As shown in Figure5B, knockdown of NDUFB6 by siRNA transfection resulted in a marked increase of cell proliferation in 786-O and 769-P. We next investigated invasion, migration, and epithelial–mesenchymal transformation (EMT)-associated gene expression. After overexpression or knockdown of NDUFB6 in 786-O and 769-P cells, invasion and migration were found to be no significant differences (Fig. S9A and B). Although knockdown of NDUFB6 induced the expression of *vimentin*, overexpression of it did not suppress the expression of *vimentin* or *ZEB1* (Fig. S9C [a] and [b]). In addition, whereas overexpression of NDUFB6 in 769-P cells induced the expression of *occludin*, knockdown of it did not suppress the expression of *occludin*. (Fig. S9C [c]). These results suggested that downregulation of NDUFB6 is not implicated in invasion or migration, but implicated in augmented cell proliferation. A flowchart to illustrate the identification process of NDUFB6 is shown in Figure S10. Furthermore, to confirm the influence of *NDUFB6* on clinical outcome, we analyzed the data of 405 cases of ccRCC obtained from TCGA Research Network. *NDUFB6* DNA copy number correlated with its mRNA expression level (Pearson correlation coefficient = 0.4113, *P *<* *0.0001) (Fig.6A). In addition, lower *NDUFB6* DNA copy number and mRNA expression level were associated with lower recurrence-free survival rates (*P *=* *0.0005 and 0.0191 by log-rank test) (Fig.6B [a] and [b]), suggesting that downregulation of NDUFB6 is implicated in poor prognosis.

## Discussion

In this study, we found that gene copy number loss at 9p24.1-p13.3 as the CNAs most significantly detected in metastatic ccRCCs. Moreover, we identified *NDUFB6* as a novel candidate gene for metastasis, according to gene copy number loss and downregulation of expression. We found that, when the *NDUFB6* gene was exogenously introduced and overexpressed in two RCC cell lines in which *NDUFB6* mRNA was downregulated, their cell proliferation was significantly suppressed. In addition, knockdown of NDUFB6 by siRNAs revealed to increase cell proliferation, suggesting that downregulation of NDUFB6 may confer a growth advantage on ccRCC with 9p loss. We suppose that metastasis is caused by multistep progression, such as moving to the metastatic site by invasion, migration, and EMT, and then proliferating at the metastatic site. In this study, we demonstrated that NDUFB6 did not affect invasion or migration activity. In addition, overexpression of *NDUFB6* did not suppress the expression of *vimentin* or *ZEB1* which are known to be increased in EMT, and its downregulation did not suppress the expression of *occludin* which is known to be decreased in EMT 16. *E-cadherin*, which is known to be suppressed in EMT, was not used because the expression levels were very low in 786-O and 769-P cells (data not shown) 16. Although we detected the statistical significance in some EMT markers, differences in expression levels were small. These results suggest that NDUFB6 is not involved in EMT phenotype. Therefore, we supposed that downregulation of NDUFB6 is implicated in cell proliferation in metastatic tumors. It has been reported that NDUFB6 is located in mitochondria and is a component of respiratory chain complex I (RCI) 17, playing an important role in the regulation of RCI including electron transfer activity 18. Furthermore, it has also been reported that RCI dysfunction may contribute to tumor progression by enhancing the metastatic potential of tumor cells 19–21. For example, silencing of the other subunits of RCI, GRIM-19, or NDUFS3, reduced RCI activity, promoted reactive oxygen species (ROS) associated with upregulation of adhesion and EMT proteins, and induced invasion in Hela cells and breast cancer cell lines 22. These findings enable us to speculate that the RCI dysfunction caused by NDUFB6 downregulation may play a role in tumor progression, including metastasis. In addition, we found that lower DNA copy number and mRNA expression levels of *NDUFB6* were associated with poorer prognosis using TCGA data. Based on the present study, we concluded that downregulation of NDUFB6 due to loss at 9p24.1-p13.3 may induce ccRCC metastasis through cell proliferation. Further study, especially for in vivo animal model will be necessary to reveal the molecular mechanisms responsible for promotion of cell proliferation due to downregulation of NDUFB6.

In 9p24.1-p13.3, there are some tumor suppressor genes including *CDKN2A* and *CDKN2B*, which have been previously reported 23–26. However, as shown in Figure S6, none of these genes were downregulated due to copy number loss. This is consistent with our previous study that not all genes show clear impact between CNA and gene expression 12. Although we could not rule out a possibility that deletions of *CDKN2A* and *CDKN2B* are responsible for development of metastasis, downregulation of these genes due to copy number loss was not detected in the present study. We speculate two possibilities: one is that these two genes are not downregulated in our cases, and the other is that expression levels of *CDKN2A* and *CDKN2B* might be already downregulated through epigenetic mechanisms in normal renal cortex. Accordingly, *CDKN2A* and *CDKN2B* were excluded as candidate genes. Surprisingly, only 2 of 58 genes showed downregulation due to copy number loss. Of course, other genes located at 9p24.1-p13.3 were indeed downregulated as shown in Figure S6. However, in most of them, expression levels were downregulated not only in tumors with copy number loss but also in those without copy number loss. Therefore, it is suggested that other tumor suppressor genes may be excluded because they are already downregulated in early stage of tumor.

Here, we identified loss at 9p24.1-p13.3 and additional CNAs including losses at 6q, 9q, 10p, 10q, 18p, and 18q as the CNAs characteristic of metastatic ccRCCs. High-resolution array CGH studies have revealed that patients whose primary tumors have 9p24.1-p21.3 loss show a poor outcome 27. Our present data support these previous findings. Furthermore, 9p24.1-p13.3 loss tended to be detected more frequently in metastases than in locally advanced ccRCCs in our present data. This result suggests that 9p24.1-p13.3 loss is a CNA associated with metastasis rather than progression. An interesting feature of our present study was that additional CNAs including losses at 6q, 9q, 10p, 10q, 18p, and 18q were found to accompany 9p24.1-p13.3 loss, and that these losses were detected in metastatic lesions irrespective of whether or not the primary lesion exhibited 9p24.1-p13.3 loss. Based on our analysis, we hypothesize that two subtypes of ccRCC might be present shown in Figure3B. One is characterized by the presence of loss at 9p and additional CNAs including losses at 6q, 9q, 10p, 10q, 18p, and 18q in the primary tumor, which tends to metastasize early and have a relatively poor prognosis (Fig.3B [b]). The other type is characterized by absence of loss at 9p in the primary tumor and tends to require a long time to metastasize after initial surgery, leading to a relatively good prognosis, since a longer time is needed to accumulate loss at 9p24.1-p13.3 and additional CNAs (Fig.3B [a]). We hypothesize that in primary lesions without 9p24.1-p13.3 loss and additional CNAs, a small subclone with 9p24.1-p13.3 loss and additional CNAs may arise during tumor development, subsequently developing and occupying the primary lesion, and finally metastasizing. Because 9p24.1-p13.3 loss and additional CNAs were found in primary tumors of patients showing early onset of metastasis after initial surgery, detection of these CNAs in primary ccRCCs may be clinically useful for prediction of early metastasis.

## Supporting Information

**Figure S1.** Clinical courses of the patients in group I after nephrectomy for primary ccRCCs. Yellow circles represent surgically resected primary ccRCCs. Red and black circles represent surgically resected metastases. Arrow, slash, *, and † represent alive, death due to cancer, data missing, and death due to other disease, respectively. The *x*-axis shows the time after nephrectomy for removal of primary ccRCC. All the primary ccRCCs and metastases indicated by red circles were subjected to array CGH.

**Figure S2.** Loss at 9p24.1-p13.3 is the most common region in chromosome 9. Summary of genomic imbalances at chromosome 9 in 20 cases of primary ccRCC and metastasis (patient group I). Green lines display copy number losses and red lines display copy number gains in each case.

**Figure S3.** Poor prognosis of patients with 9p24.1-p13.3 loss in primary ccRCCs of both patient group I and II. (A) Recurrence-free survival rates (shown by Kaplan–Meier curve) for 13 patients with 9p24.1-p13.3 loss in primary ccRCCs of both patient group I and II, excluding nine patients with stage IV disease, were lower than those for 25 patients without 9p24.1-p13.3 loss, excluding three patients with stage IV (*P *=* *0.0032 determined by log-rank test). (B) Cancer-specific survival rates (shown by Kaplan–Meier curve) for 22 patients with 9p24.1-p13.3 loss in primary ccRCCs of both patient group I and II were lower than those for 28 patients without 9p24.1-p13.3 loss (*P *=* *0.0012 determined by log-rank test).

**Figure S4.** Clinical outcome is not different regardless of administration of therapeutic agents. Cancer-specific survival rates (shown by Kaplan–Meier curve) for 10 patients who were administrated therapeutic agents for metastatic lesions before surgical resection and those for 10 patients who were not. No significant differences were found between them. NS, not significant.

**Figure S5.** Genomic profiles of metastatic ccRCCs which are administrated therapeutic agents or not. Genome-wide averaged frequencies of genomic imbalances in 10 cases (patient group I) of metastatic ccRCC which were administrated therapeutic agents before surgical resection (A) and those in 10 cases which were not (B). Oligonucleotide probes are shown in order within each chromosome from chromosomes 1 to 22. The frequencies (%) of gains (positive axis) and losses (negative axis) are shown for each probe.

**Figure S6.** Correlation between mRNA expression and CNA status. Expression levels of the 53 annotated genes at 9p24.1-p13.3 between 5,536,660 and 32,972,853 base pairs except for *NDUFB6*,*LRRC19*, and three genes whose mRNA expression levels were undetectable. None of these genes were downregulated due to copy number loss. Quantitative RT-PCR analyses of 30 primary ccRCCs and 19 samples of normal control tissue (patient group II) were performed. The *y*-axis displays the expression level (log_2_) normalized by the median expression level for the 19 normal control samples. **P *<* *0.05, ***P *<* *0.01, ****P *<* *0.001, and *****P *<* *0.0001 determined by Kruskal–Wallis test and Steel–Dwass test as a post hoc test. NS, not significant.

**Figure S7.***NDUFB6* and *LRRC19* are downregulated in 786-O and 769-P cells. Quantitative RT-PCR analysis of 786-O cells, 769-P cells, and 19 samples of normal control tissue (patient group II) for expression of *NDUFB6* (A) and *LRRC19* (B). The *y*-axis displays the expression level (log_2_) normalized by the median expression level for the 19 normal controls.

**Figure S8.** Apoptosis is not induced by overexpression of *NDUFB6* and *LRRC19* in RCC cell lines. The 786-O cells and 769-P cells were incubated for 72 h after transduction of Lv-Control, Lv-NDUFB6, or Lv-LRRC19, and subsequent cytoplasmic oligonucleosomal fragmentation was measured using the Cell Death Detection ELISA^PLUS^ assay, as described in Data S1. The *y*-axis displays the absorbance at 405 nm (reference wave length 492 nm). The data are expressed as mean ± SEM of quadruplicate determinations and differences were analyzed by Mann–Whitney *U* test.

**Figure S9.** Influence of overexpression and knockdown of NDUFB6 on invasion, cell migration, and EMT phenotype. (A) Invasion is neither influenced by overexpression nor knockdown of NDUFB6. Lentivirally transduced or siRNA-transfected 786-O and 769-P cells were subjected to an invasion assay, as described in Data S1. The *y*-axis displays the invaded cells detected as absorbance at 590 nm. The data are expressed as mean ± SEM of triplicate determinations and differences were analyzed by Mann–Whitney *U* test. Two independent experiments were performed and representative data are shown. (B) Cell migration is neither influenced by overexpression nor knockdown of NDUFB6. Lentivirally transduced or siRNA-transfected 786-O and 769-P cells were subjected to a migration assay, as described in Data S1. The *y*-axis displays the migrated cells. The data are expressed as mean ± SEM of duplicate determinations and differences were analyzed by Mann–Whitney *U* test. (C) Influence of overexpression and knockdown of NDUFB6 on expression of EMT-associated genes. Total RNA was extracted from lentivirally transduced or siRNA-transfected 786-O and 769-P. Expression levels of *vimentin* (a), *ZEB1* (b), and *occludin* (c) known as EMT-associated genes were analyzed by qRT-PCR. The *y*-axis displays the mRNA expression levels (fold change) of the genes. The data are expressed as mean ± SEM of quadruplicate determinations, differences were analyzed by Mann–Whitney *U* test. **P *<* *0.05 determined by Mann–Whitney *U* test.

**Figure S10.** The process of identification of *NDUFB6* as a novel candidate gene for metastasis. 9p24.1-p13.3 loss was identified by array CGH and analysis of clinical prognosis using 20 cases of primary ccRCC and metastasis (patient group I). Two downregulated genes due to copy number loss were identified by array CGH and quantitative RT-PCR using 30 cases of primary ccRCC (patient group II). *NDUFB6* was identified as a novel candidate gene for metastasis by proliferation assay using RCC cell lines.

**Table S1.** Clinical and pathological characteristics of the patients (groups I and II).

**Table S2.** CNAs detected more frequently in primary ccRCCs with 9p24.1-p13.3 loss than in those without.

**Table S3.** CNAs frequently detected in metastases derived from primary ccRCCs without 9p24.1-p13.3 loss.

**Table S4.** Losses at 18p and 18q detected in metastases derived from primary ccRCCs without 9p24.1-p13.3 loss.

**Table S5.** Relative expression of genes located at 9p24.1-p13.3.

**Table S6.** Copy number alterations of 58 genes located at 9p24.1-p13.3 in patient group II.
