# Supplementary figures and images for: Downregulation of NDUFB6 due to 9p24.1-p13.3 loss is implicated in metastatic clear cell renal cell carcinoma
Source: Cancer Med. 2014 Oct 15;4(1):112–24. doi: 10.1002/cam4.351 (PMC4312125; doi:10.1002/cam4.351)

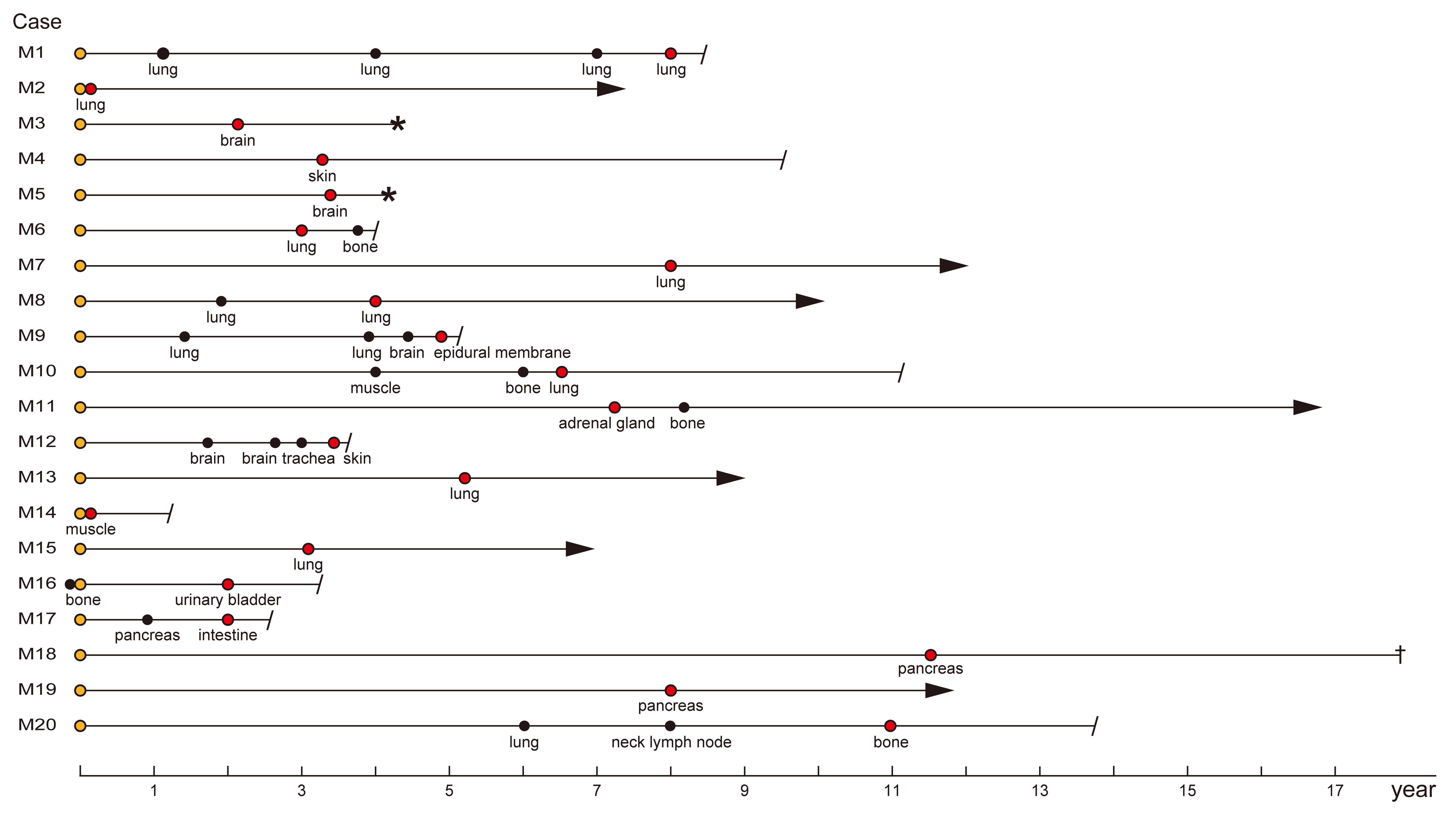

Supplement: Supplementary file 1 [file cam40004-0112-sd1.tif]

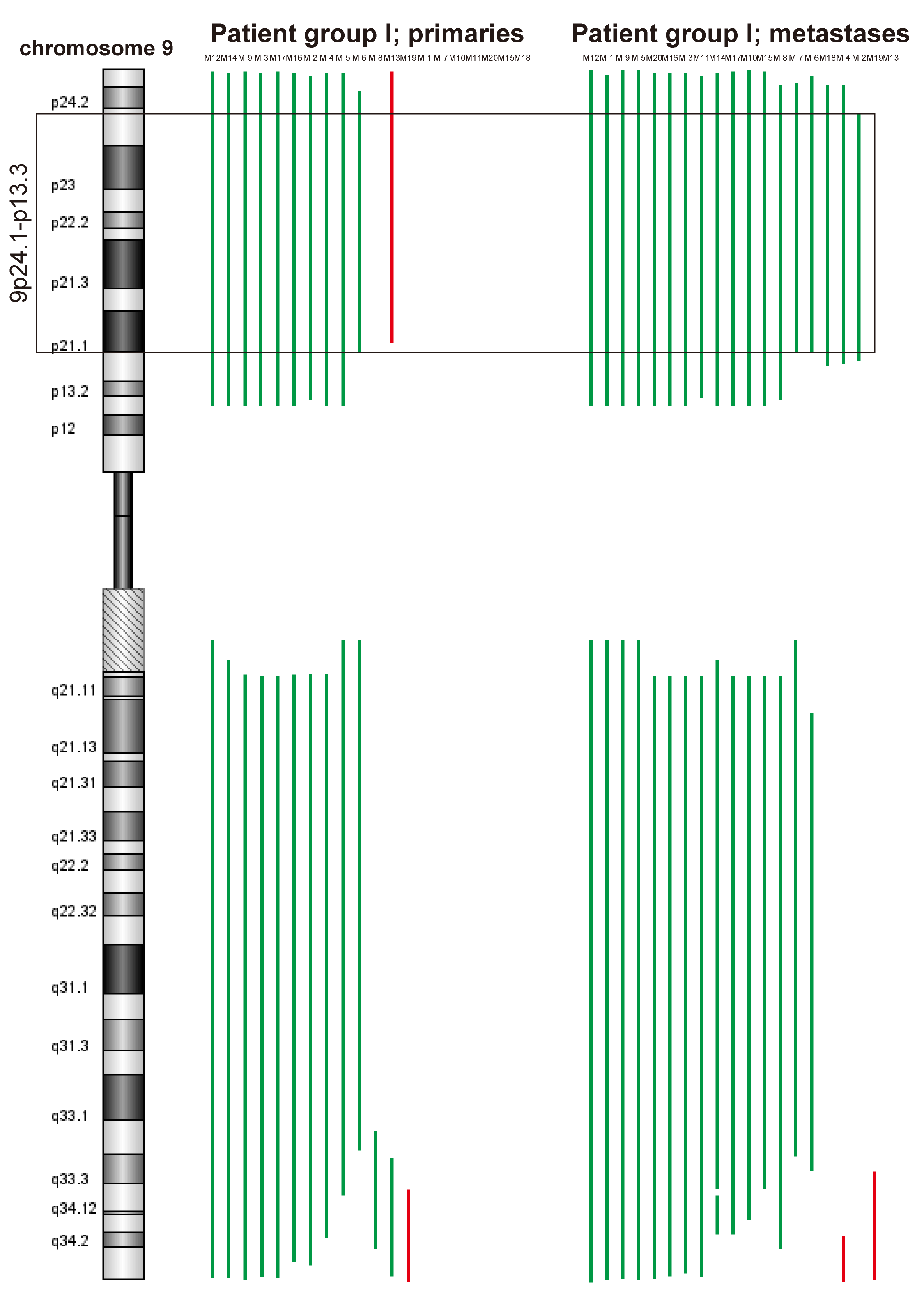

Supplement: Supplementary file 2 [file cam40004-0112-sd2.tif]

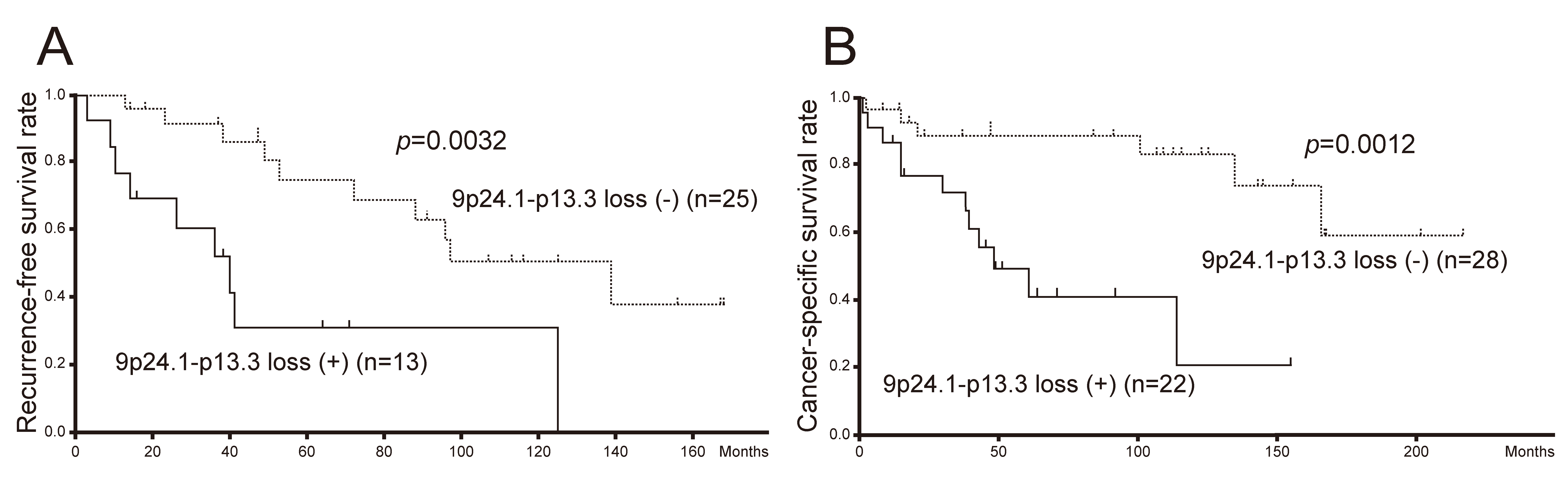

Supplement: Supplementary file 3 [file cam40004-0112-sd3.tif]

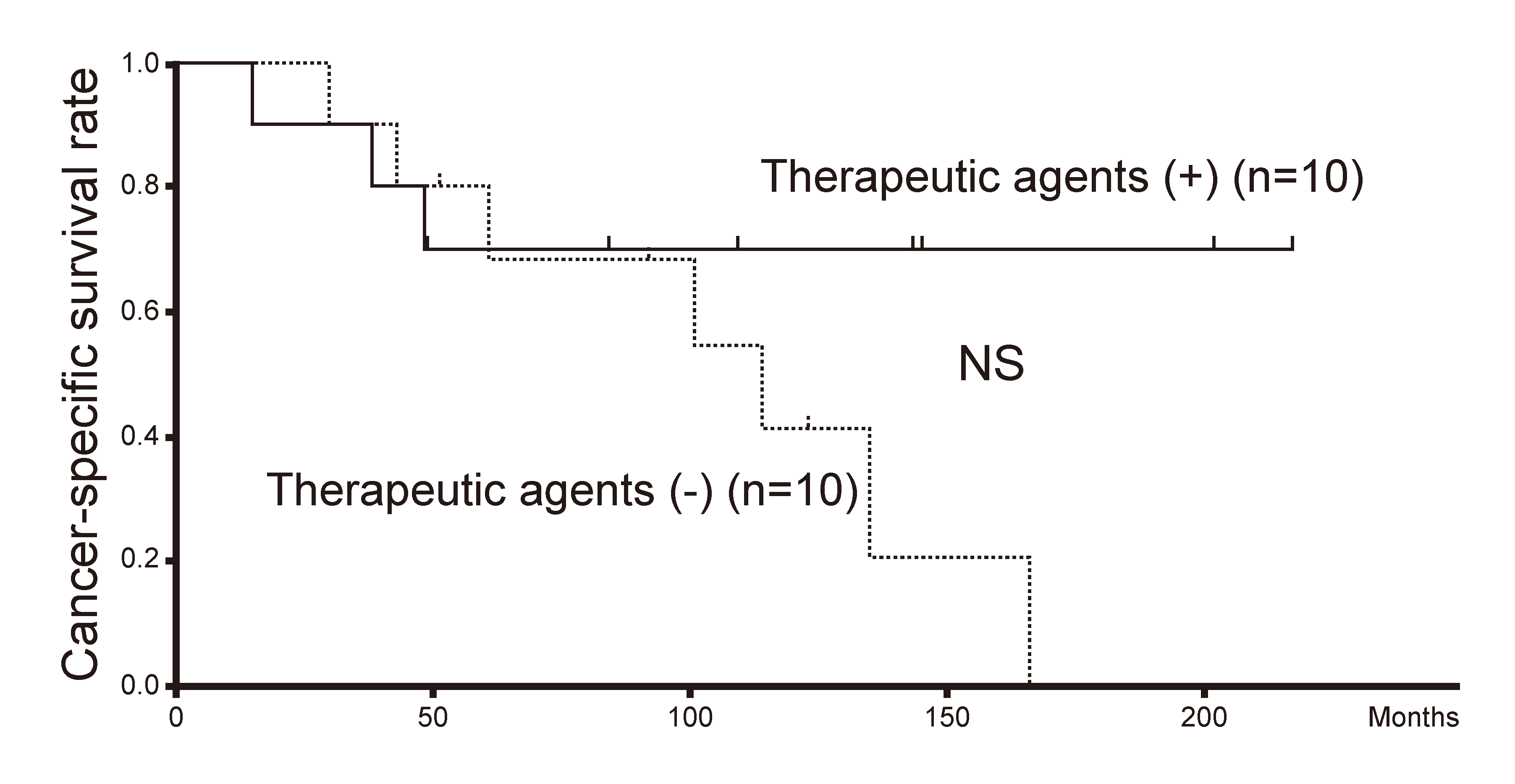

Supplement: Supplementary file 4 [file cam40004-0112-sd4.tif]

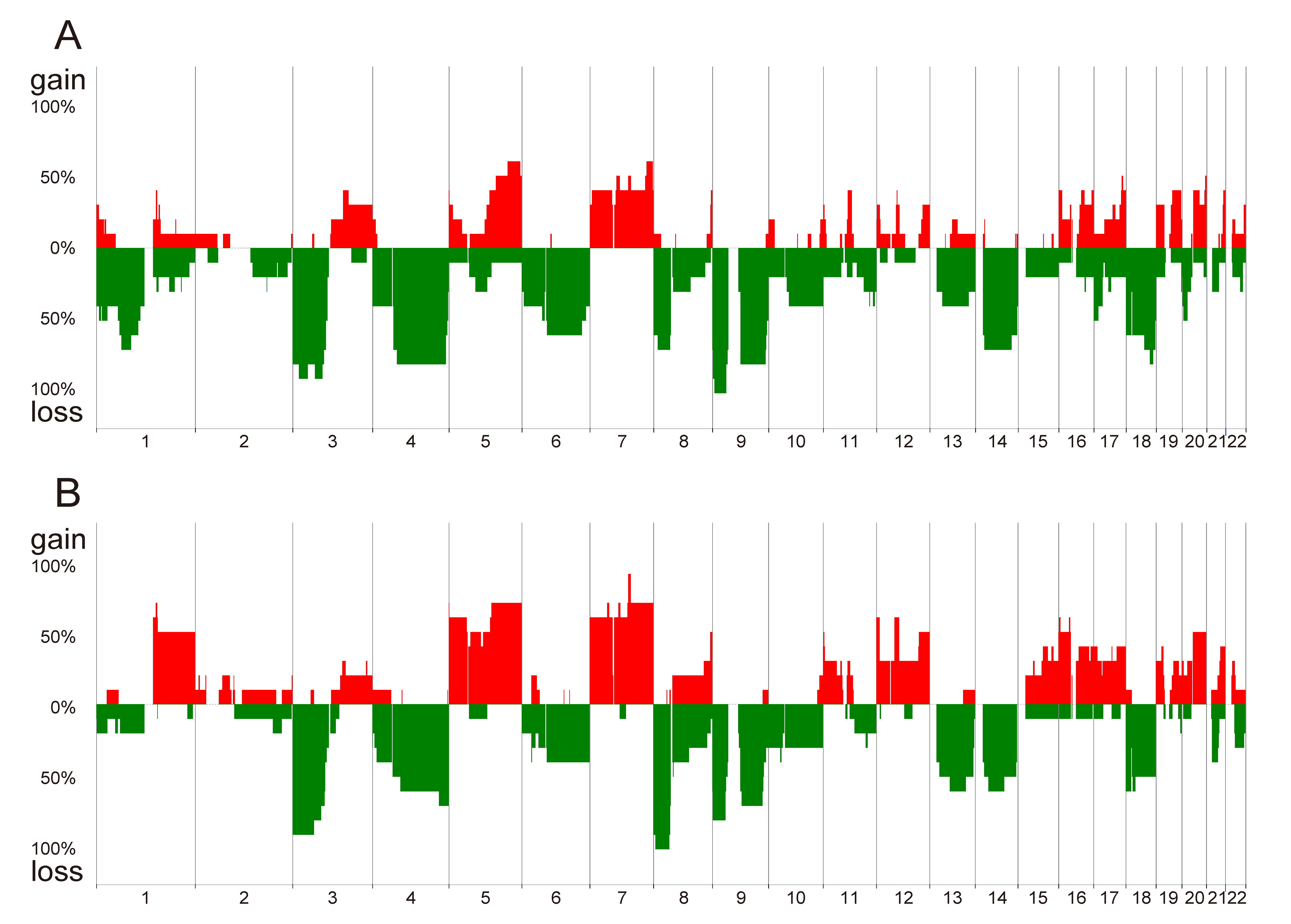

Supplement: Supplementary file 5 [file cam40004-0112-sd5.tif]

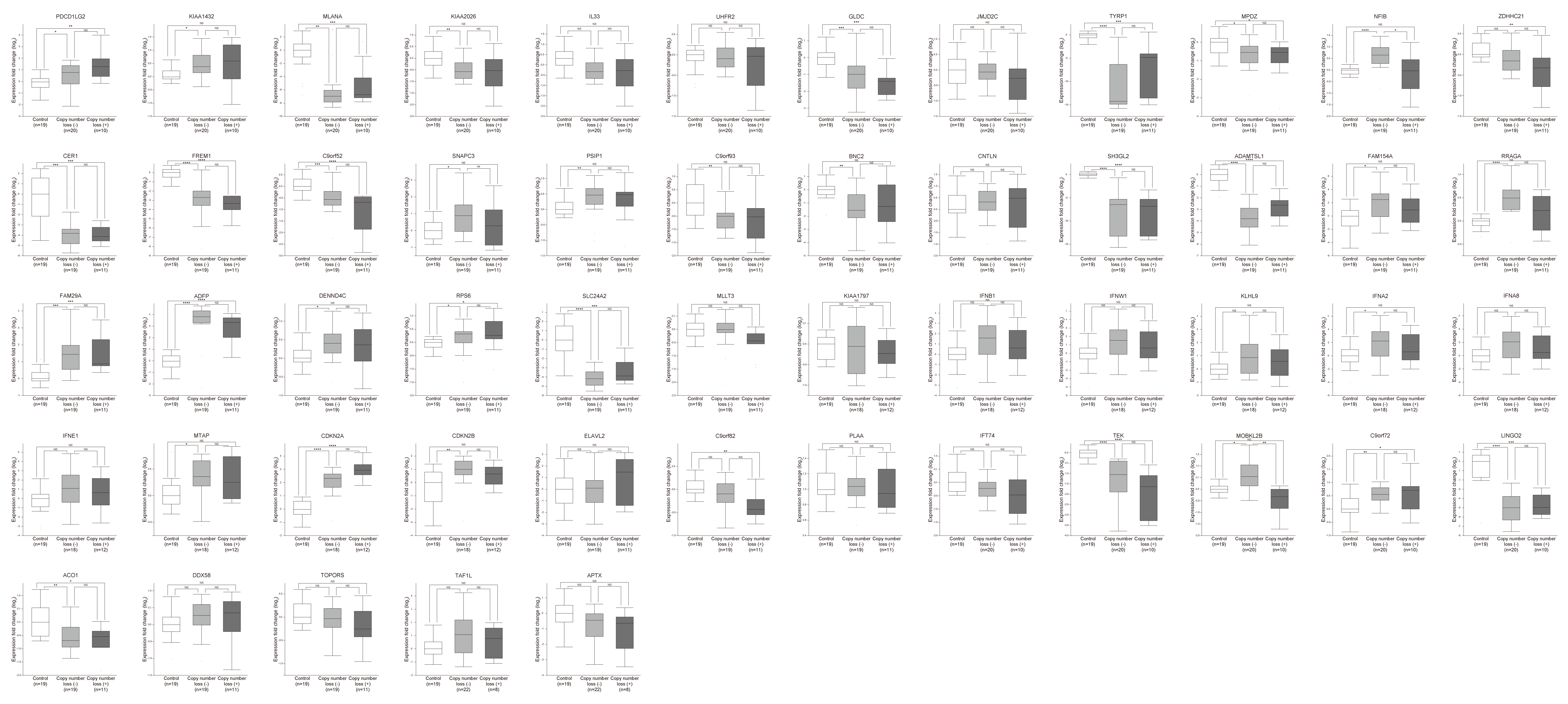

Supplement: Supplementary file 6 [file cam40004-0112-sd6.tif]

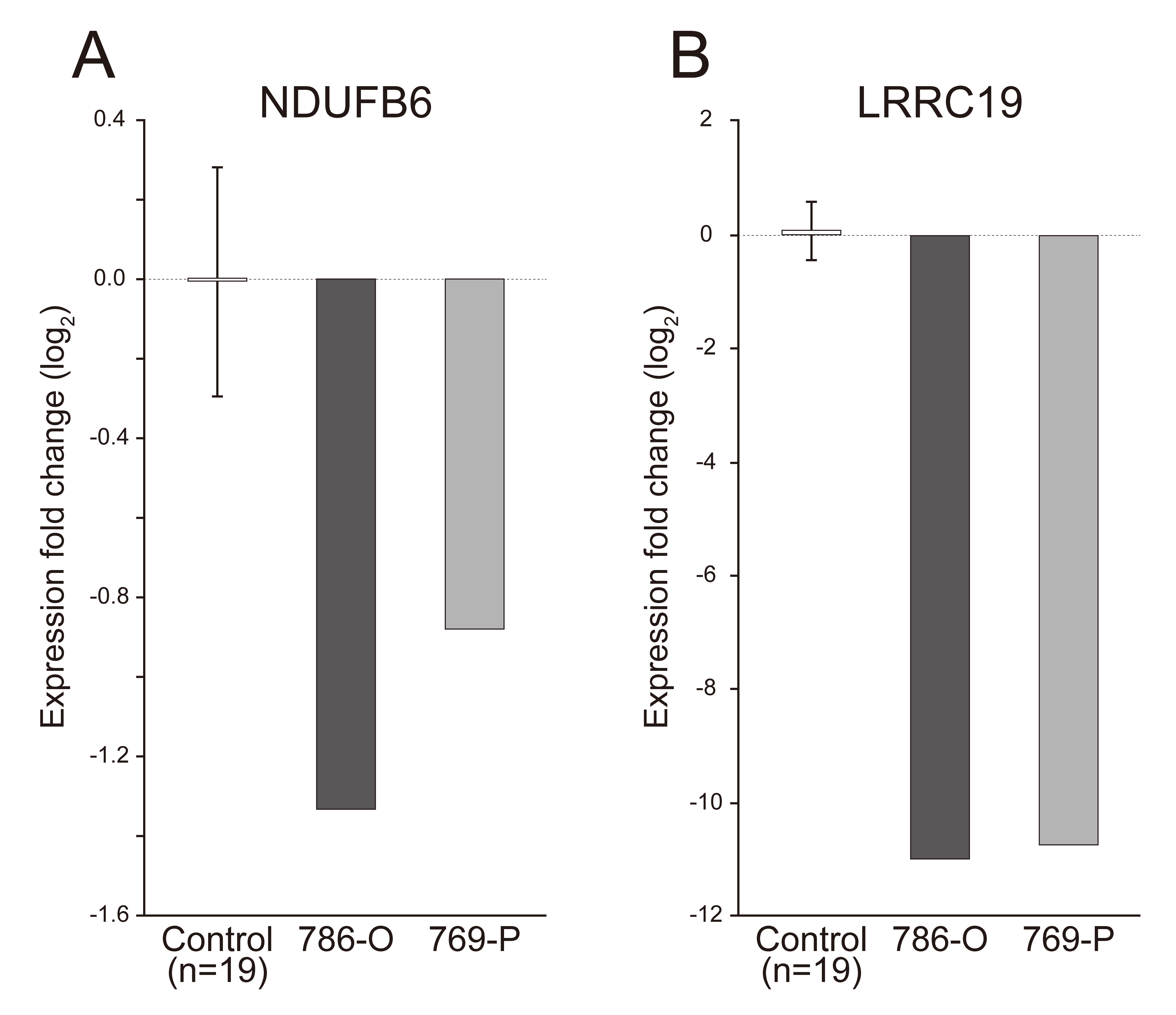

Supplement: Supplementary file 7 [file cam40004-0112-sd7.tif]

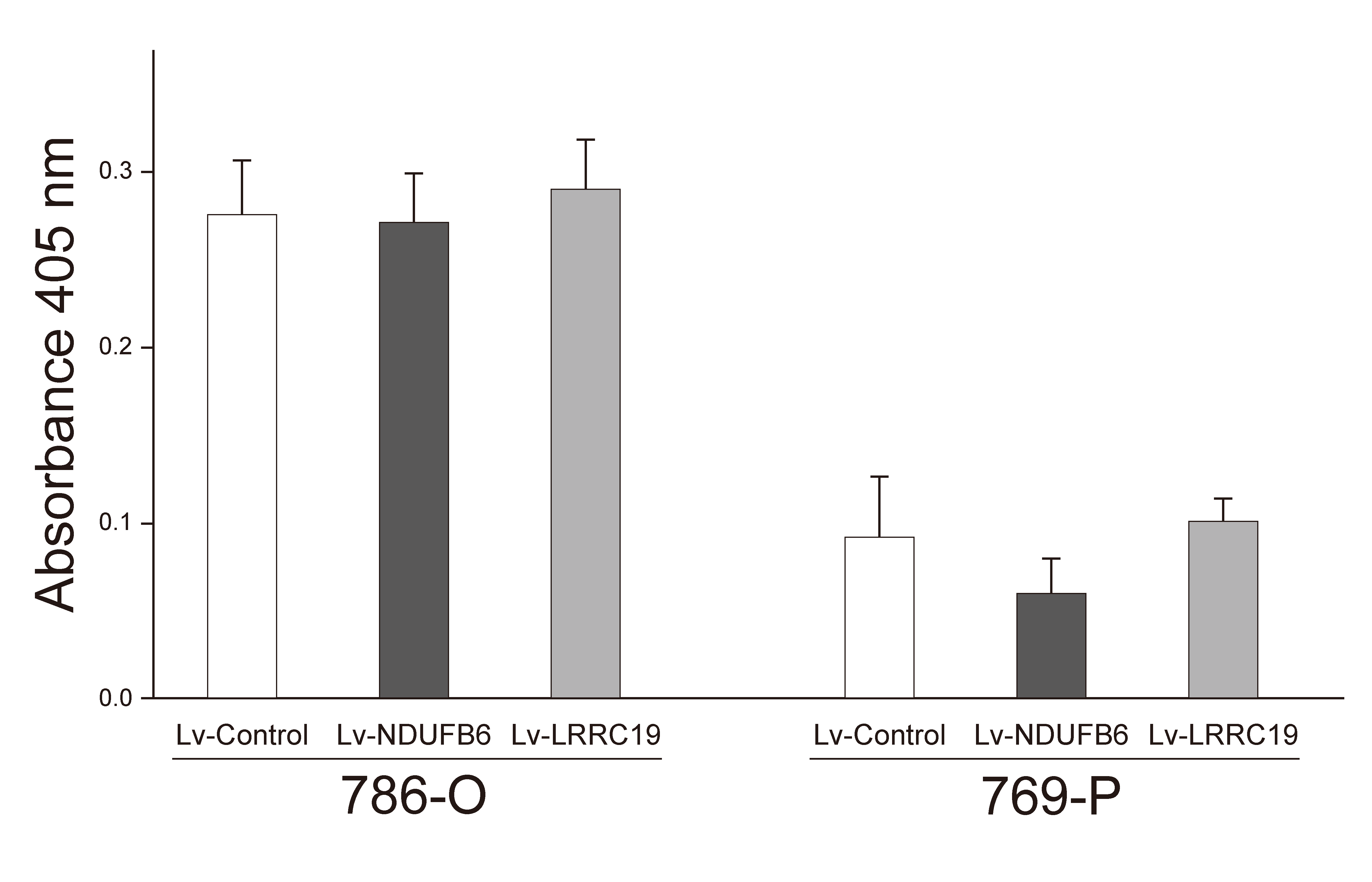

Supplement: Supplementary file 8 [file cam40004-0112-sd8.tif]

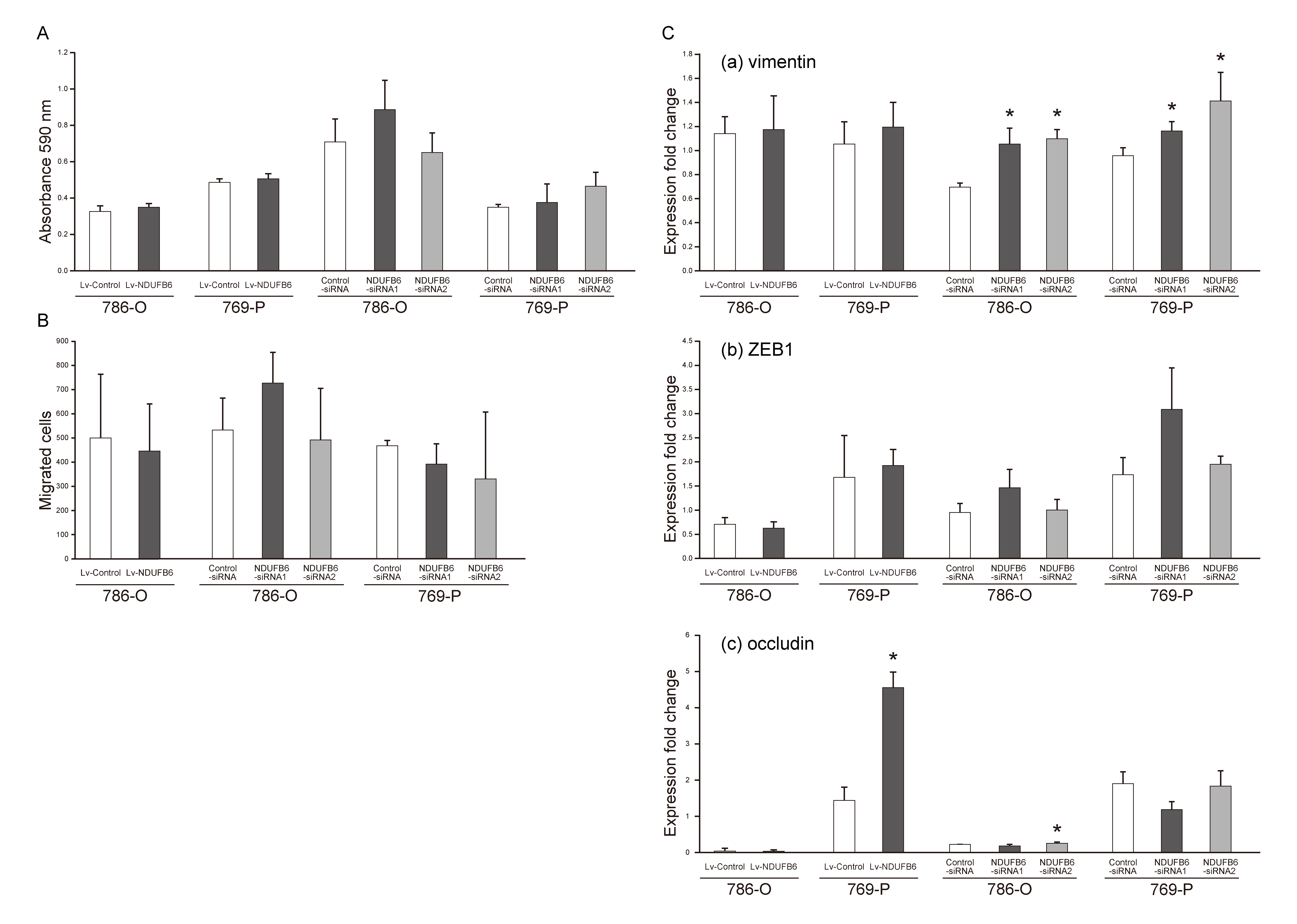

Supplement: Supplementary file 9 [file cam40004-0112-sd9.tif]

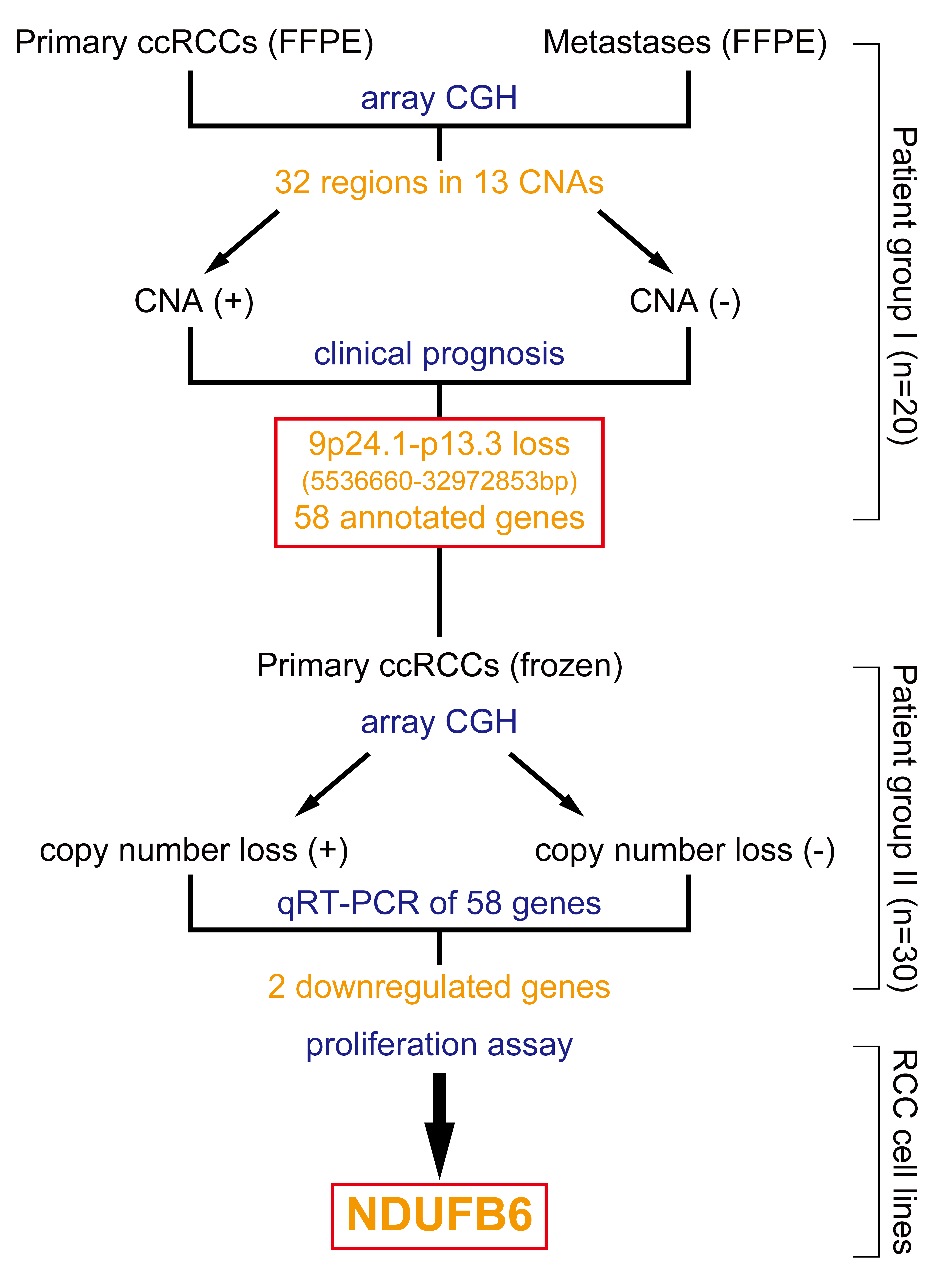

Supplement: Supplementary file 10 [file cam40004-0112-sd10.tif]
