# Supplementary material for: Downregulation of NDUFB6 due to 9p24.1-p13.3 loss is implicated in metastatic clear cell renal cell carcinoma
Source: Cancer Med. 2014 Oct 15;4(1):112–24. doi: 10.1002/cam4.351 (PMC4312125; doi:10.1002/cam4.351)
Supplement: Supplementary file 17 [file cam40004-0112-sd17.doc]

**Supplementary Materials & Methods**

**Array CGH and data analysis**

Array CGH was performed using the Agilent Whole Human Genome 4x44 K Oligo Micro Array Kit (Agilent Technologies) in accordance with the manufacturer’s instructions. Microarray images were analyzed using FEATURE EXTRACTION v.9.1.3.1, v.9.5.1.1 or v.9.5.3.1 (Agilent Technologies) with linear normalization (protocol CGH-v4_91 or CGH-v4_95_Feb07), and the resulting data were subsequently imported into the DNA Analytics v.4.0.81 software package (Agilent Technologies). Following normalization of the raw data, the log_2_ ratio of Cy5 (tumor) to Cy3 (control) was calculated. Aberrant regions were determined by the Aberration Detection Method-2 algorithm at a threshold of 6.0 in DNA Analytics. To detect gains and losses, we set the values of parameters for the aberration filters as follows: minimum number of probes in region 2, minimum absolute average log_2_ ratio for region 0.10, maximum number of aberrant regions 10000, and percentage penetrance per feature 0. The data generated by probes mapped to the X and Y chromosomes were eliminated. The data obtained in the array CGH analysis are available on the GEO database (http://www.ncbi.nlm.nih.gov/geo/; accession number GSE 43477). Frequencies of CNAs between the matched pairs of tumor groups and those between the unmatched groups were compared by McNemar test and Fisher’s exact test, respectively. CNAs with *p* values of less than 0.05 and a false discovery rate (FDR) of less than 0.3 after correction for multiple testing were considered to be statistically significant. The multiple testing correction methodology is described in supplementary materials and methods: Multiple testing correction.

**Multiple testing correction**

To account for the effect of multiple testing on the statistical significance of the CNA, we computed the FDR. To quantify the difference of CNAs between the tumors, we performed the McNemar test for comparing the CNAs between the primary ccRCCs and metastases derived from the same cases, and Fisher’s exact test for comparing the CNAs between the tumors derived from different cases. To compute the FDRs, we used the label permutation procedure. To estimate the family-wise null distributions of the set of McNemar *p*-values and Fisher exact *p*-values, we randomly shuffled the labels and computed the set of null McNemar *p*-values and Fisher exact *p*-values. We repeated this operation 10,000 times with different random seeds. Using the estimated family-wise null distributions for a set of McNemar *p*-values and Fisher exact *p*-values, we computed the FDR of each CNA.

**Apoptosis assay**

After the infection of Lv-NDUFB6, Lv-LRRC19 or Lv-Control to 786-O and 769-P cells in a 96-well plate, they were cultured for 72 h. DNA fragmentation was detected in a 96-well format using Cell Death Detection ELISA^PLUS^ (Roche Applied Science, Mannheim, Germany) in accordance with the manufacturer’s instructions. Each condition was reproduced in quadruplicate.

**Invasion assay**

Invasion assay was performed using Transwell**®** polycarbonate membranes (6.5 mm diameter, 8.0 μm pore size, #3422, Corning Inc., Corning, NY, USA) in a modified Boyden chamber. Before the assay, the lower surface of the membrane was coated with 1 μg of human fibronectin (Roche Diagnostics) and air-dried overnight. The upper surface was coated with 5 μg of Matrigel (BD, Franklin Lakes, NJ, USA) and air-dried for 3 h. Lentivirally transduced or siRNA transfected 786-O (2 x10^4^) and 769-P (6 x10^4^) cells in 100 μl of medium with 0.1% BSA were seeded into the inner chamber, and 600 μl of medium with 0.1% BSA was added to the lower chamber. Subsequently, the plate was incubated at 37°C in 5% CO_2_ for 24 h and fixed with 30% methanol for 1 min. After washing of the non-invaded cells, the membranes were stained with crystal violet (Wako, Osaka, Japan), and eluted with 30% acetic acid for measurement of the absorbance at 590 nm using a fluorescence reader (Multiskan GO) (Thermo Scientific). Each condition was reproduced in triplicate, and the experiment was performed twice.

**Migration assay**

Lentivirally transduced or siRNA transfected cells were seeded at 2 x 10^4^ cells per well into Oris^TM^ Cell Migration Assay - Collagen I Coated 96-well plate. After 4 h, the plugs were removed and cells were allowed to migrate for 16 h. Then, cells were stained with Calcein for 1 h, and total number of migrated cells were counted. Each condition was reproduced in duplicate.

**EMT associated gene expression analysis**

Total RNA was extracted from lentivirally transduced or siRNA transfected cells using an RNeasy mini kit (Qiagen). Expression levels of *vimentin*, *ZEB1* and *occludin* known as EMT associated genes were analyzed by qRT-PCR with a Universal probe library (Roche Diagnostics) and a LightCycler 480 probe master (Roche Diagnostics) by the Taqman method, as described in materials and methods. Differences were analyzed by Mann-Whitney *U* test. Each condition was reproduced in quadruplicate.
